# Less Favorable Lipid Profile and Higher Prevalence of Thyroid Antibodies in Women of Reproductive Age with High-Normal TSH—Retrospective Study

**DOI:** 10.3390/ijerph17062122

**Published:** 2020-03-23

**Authors:** Małgorzata Karbownik-Lewińska, Jan Stępniak, Anna Żurawska, Andrzej Lewiński

**Affiliations:** 1 Department of Oncological Endocrinology, Medical University of Lodz, 90-752 Lodz, Poland; jan.stepniak@umed.lodz.pl (J.S.); anzurawska@gmail.com (A.Ż.); 2 Polish Mother’s Memorial Hospital—Research Institute, 93-338 Lodz, Poland; alewin@csk.umed.lodz.pl; 3 Department of Endocrinology and Metabolic Diseases, Medical University of Lodz, 93-338 Lodz, Poland

**Keywords:** thyroid tests, TSH < 2.5 mIU/L, euthyroid, reproductive age, lipid profile

## Abstract

High-normal TSH can be associated with metabolic abnormalities and infertility. Aims of this study are to analyze retrospectively if routinely measured blood laboratory and anthropometric parameters differ between women of reproductive age with TSH < 2.5 mIU/L and with TSH ≥ 2.5 mIU/L. Retrospective analysis was performed in 466 female inpatients, aged 13–51, hospitalized in an endocrine department. The group of 280 patients with normal thyroid tests (TSH 0.27-4.2 mIU/L; normal FT3 and FT4) was selected and it was divided into two subgroups, i.e., with TSH < 2.5 mIU/L and TSH ≥ 2.5 mIU/L (n = 66; 23.6%). After excluding patients on L-thyroxine treatment (n = 240), those with TSH ≥ 2.5 mIU/L constituted 22.92% (n = 55). In the group of 280 patients with normal thyroid tests, an abnormally high concentration of triglycerides and an abnormally low HDLC/cholesterol ratio occurred more frequently in women with TSH ≥ 2.5 mIU/L than those with TSH < 2.5 mIU/L (17% vs. 7%, *p* = 0.017; 14% vs. 5%, *p* = 0.015, respectively). Increased concentration of thyroid antibodies, i.e., TPOAb, occurred more frequently in patients with TSH ≥ 2.5 mIU/L than those with TSH < 2.5 mIU/L (27% vs. 9%, *p* = 0.001). The same differences were found in the group of 240 patients after excluding those on L-thyroxine treatment. Blood lymphocyte concentration was the only independent linear parameter associated with TSH ≥ 2.5 mIU/L (OR = 1.551, *p* = 0.024) but only in the group of 280 patients with normal thyroid tests. TSH concentration correlated positively with blood lymphocyte (r = 0.129, *p* = 0.031) and TPOAb (r = 0.177, *p* = 0.005) concentrations but only in the group of 280 patients with normal thyroid tests. Less favorable lipid profiles and a higher prevalence of thyroid antibodies in women of reproductive age with high-normal TSH suggests that L-thyroxine treatment should be considered in such patients. The significance of a positive association between high-normal TSH and blood lymphocytes requires further evaluation.

## 1. Introduction

Thyroid tests depend on age. TSH increases with age and is thus found in lower normal ranges in younger subjects [1,2]. It is generally accepted that optimal thyroid tests are important for reproduction (e.g., Korevaar et al. [3]). It has been recommended for a long time that TSH levels below 2.5 mIU/L should be maintained during preconception and pregnancy and still numerous authorities support such a point of view [4,5,6]. In agreement with this, lower TSH should be expected in most healthy non-pregnant population of childbearing age. According to the most recent recommendation by the American Thyroid Association (2017) [7], as high TSH as 4.0 mIU/L is accepted as the upper limit during pregnancy. Although a small percentage of the healthy population may have TSH in the upper normal range, and this relates also to women of childbearing age, such high-normal TSH at preconception and during pregnancy always requires particular attention in individuals.

It has been documented in several studies that high-normal TSH is associated with some adverse effects concerning reproduction and with certain abnormalities in metabolic processes. Concerning the former, either preconception (e.g., Chen et al. [8]) or first trimester high-normal TSH (e.g., Hernandez et al., Kianpour et al. [9,10]) are associated with both infertility and unfavorable pregnancy outcomes. Concerning other undesired effects, the association between high-normal TSH and an abnormal lipid profile was described, for example, in euthyroid non-smokers with newly diagnosed heart disease [11] and in women of childbearing age [12]. These changes in lipid profile found in cases of high-normal TSH are similar to those frequently observed in individuals, children included, with subclinical hypothyroidism (defined as TSH above the normal range) [13]. Of great importance is our last finding showing that TSH ≥ 2.5 mIU/L is associated with the increased oxidative damage to membrane lipids in women of childbearing age with normal thyroid tests [14]. At the same time and expectedly, these patients had a worse lipid profile [14]. We have also recently observed that TSH ≥ 2.5 mIU/L is associated with the lower level of mannan-binding Lectin (a component of the lectin pathway of the complement system) in women of childbearing age [15].

Taking into account the above observations, new evidence is required to document that high-normal TSH in women of childbearing age may be associated with some pathological processes and, therefore, should be treated as abnormal.

The aim of this study was to retrospectively analyze if routinely measured blood laboratory parameters as well as anthropometric parameters differ between women of reproductive age with TSH < 2.5 mIU/L and those with TSH ≥ 2.5 mIU/L.

## 2. Materials and Methods

The procedures used in the study were approved by the Ethical Committee of the Polish Mother’s Memorial Hospital—Research Institute, Poland [No. 40/2018].

Retrospective analysis was performed in 466 female inpatients, aged 13–51, hospitalized in 2016 and 2017 with a suspicion of different endocrine diseases (such as polycystic ovary syndrome, prolactinoma, partial hypopituitarism, etc.), thyroid dysfunction included, in the Department of Endocrinology and Metabolic Diseases, Medical University of Lodz, Poland. Only patients, in whom endocrine entities (apart from thyroid dysfunction) were not confirmed were enrolled into this retrospective analysis.

The following laboratory parameters were measured: total cholesterol, HDL cholesterol (HDLC), LDL cholesterol (LDLC), HDLC/cholesterol ratio, triglycerides (TGs), glucose, insulin, insulin resistance index (IRI), C-reactive protein (CRP), erythrocyte sedimentation rate (ESR), vitamin D, iron, and complete blood count, i.e., red blood cells (RBC), hemoglobin (Hgb), white blood cells (WBC), neutrophils, lymphocytes, and platelets. Anthropometric measurements included body height, body mass, and BMI.

Thyroid tests, i.e., TSH, free thyroxine (FT4), and free triiodothyronine (FT3), and thyroid antibodies, i.e., thyroid peroxidase antibodies (TPOAbs), thyroglobulin antibodies (TgAbs), and TSH receptor antibodies (TSHRAbs), were evaluated in morning blood samples from all inpatients. Two hundred and eighty patients with normal thyroid tests (TSH 0.27–4.2 mIU/L; FT4 0.93–1.7 ng/dl; FT3 2.6–4.4 pg/mL) were selected. They were divided into two subgroups, i.e., 214 subjects with TSH < 2.5 mIU/L (Controls), and 66 subjects with TSH ≥ 2.5 mIU/L, the latter constituting 23.6% of the whole sample examined. No statistically significant differences were found between subgroups concerning age and body mass index (BMI) when evaluated by Student’s unpaired *t*-test (Table 1).

Thyroid antibodies were abnormal in 42 patients: only TPOAbs were abnormal (≥ 34 IU/mL) in 15 patients; only TgAbs were abnormal (≥ 115 IU/mL) in 10 patients; both TPOAbs and TgAbs were abnormal in 16 patients, and all three thyroid antibodies were abnormal (TSHRAb ≥ 1.75 IU/L) in one patient. Forty patients were on L-thyroxine replacement therapy (25–150 µg daily) due to previously diagnosed hypothyroidism; two of them were diagnosed after subtotal thyroidectomy due to either non-toxic or toxic nodular goiter, and both were in the group with TSH < 2.5 mIU/L.

All patients enrolled into retrospective analysis neither suffered from any entity affecting thyroid tests, nor obtained medication which could have interfered with laboratory methods to measure thyroid tests. None of them obtained medication that can affect the lipid profile.

Exclusion criteria constituted exposure to ionizing radiation or to any other potential prooxidative agent, alcohol consumption, cigarette smoking, and any diagnosed acute or chronic disease (any critical illness included), apart from diagnosed and properly treated thyroid dysfunction.

### 2.1. Laboratory Parameters

Thyroid tests [TSH, FT4, FT3] and thyroid antibodies (i.e., TPOAbs, TgAbs, and TSHRAbs) were measured in blood serum with an immunochemiluminescent method (Cobas e-601; Roche Diagnostics). Other laboratory parameters were measured in the blood with standard methods (Vitros 5,1; Johnson&Johnson, High Wycombe, UK).

### 2.2. Statistical analysis

The data were statistically analyzed using Student’s unpaired *t*-test. The results are presented as means ± SEM. Univariate and multivariate logistic regression analyses were used to determine which continuous variable might have been associated with TSH ≥ 2.5 mIU/L. For the evaluation of the correlation among particular parameters, Pearson’s correlation coefficient was used. The two-sided ratio comparison test was used to evaluate the frequency of events. Statistical significance was determined at the level of *p* < 0.05.

## 3. Results

Patients with TSH ≥ 2.5 mIU/L constituted 23.6% (n = 66) of all patients with normal thyroid tests (n = 280) or 22.92% (n = 55) of patients after excluding L-thyroxine treatment (n = 240). Mean values of all linear parameters in a subgroup with TSH < 2.5 mIU/L and in the subgroup with TSH ≥ 2.5 mIU/L, evaluated in the group of 280 inpatients with normal thyroid tests are presented in Table 1. The two subgroups considered were equal concerning such parameters as age, body mass, height, and BMI, and concerning most blood parameters apart from two. The two parameters which differ between subgroups and were lower in patients with TSH < 2.5 mIU/L were TPOAb concentrations and lymphocyte concentrations (Table 1). It should be stressed, however, that the absolute number of lymphocytes was not above the upper reference range (<6.5 x 10^3^/μL) in all patients. In the group of 240 inpatients (after excluding 40 patients on L-thyroxine replacement), no statistical differences were found between the subgroups with TSH < 2.5 mIU/L and those with TSH ≥ 2.5 mIU/L concerning all linear parameters (data not shown).

**Figure 1:**
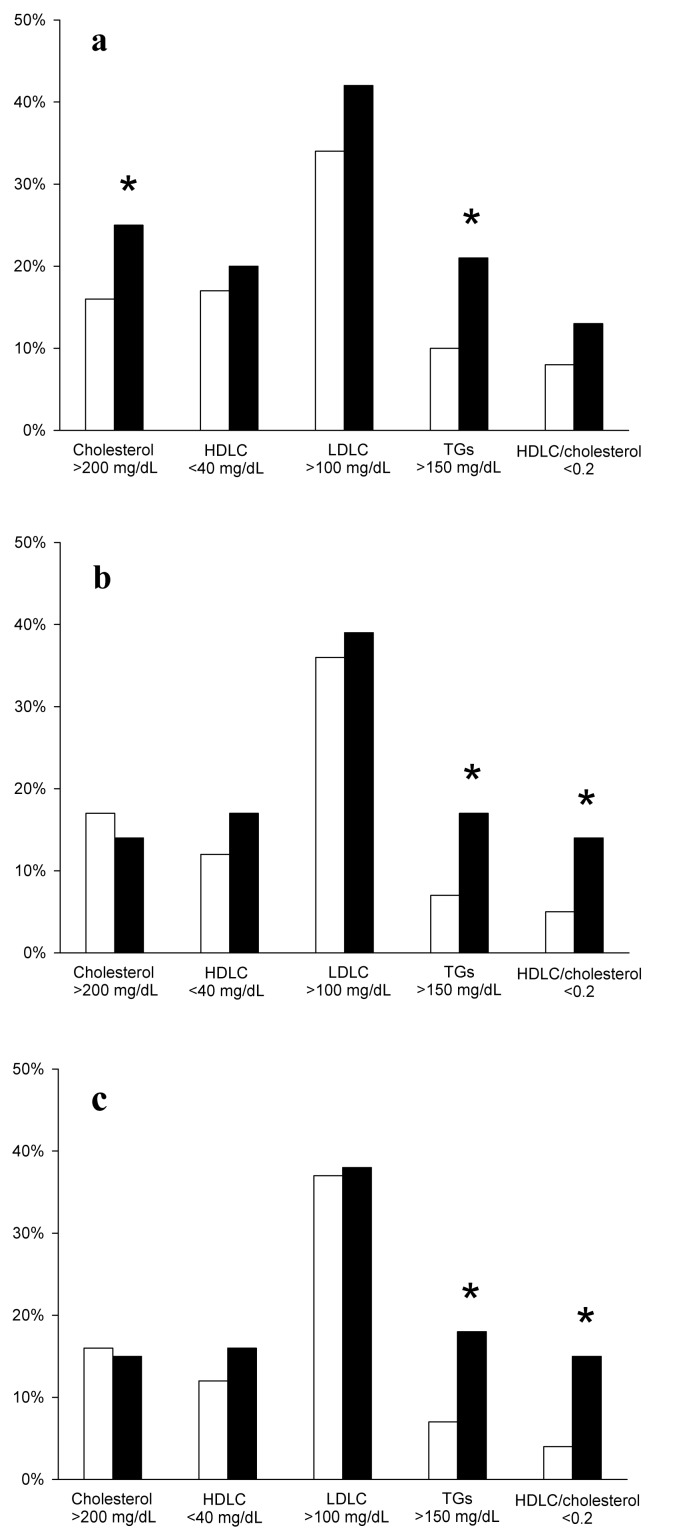
Percentage of abnormal values of particular parameters of lipid profile in patients with TSH < 2.5 mIU/L (white bars) and in patients with TSH ≥ 2.5 mIU/L (black bars), evaluated in the whole group of 466 inpatients (**a**), in the group of 280 inpatients with normal thyroid tests (**b**) or in the group of 240 inpatients (after excluding 40 patients on L-thyroxine treatment) (**c**). Statistical evaluation was performed by the two-sided ratio comparison test. *p < 0.05 vs. patients with TSH < 2.5 mIU/L.

The percentage of abnormal lipid profiles differ between subgroups concerning TGs concentrations and HDL/cholesterol ratio. Either in the group of 280 inpatients with normal thyroid tests (Figure 1b) or in the 240 inpatients (after excluding patients treated with L-thyroxine, Figure 1c), an abnormally high concentration of triglycerides and an abnormally low HDLC/cholesterol ratio occurred more frequently in subjects with TSH ≥ 2.5 mIU/L. In the whole group of 466 inpatients (Figure 1a), a statistically significant difference was additionally found concerning cholesterol concentration.

Concerning thyroid antibodies, increased concentrations of TPOAbs and TgAbs occurred more frequently in subjects with TSH ≥ 2.5 mIU/L, when the whole group (n = 466) of patients was considered (Table 2a). However, in the group of 280 inpatients with normal thyroid tests or after excluding L-thyroxine replacement (n = 240), only TPOAb concentrations occurred more frequently in subjects with TSH ≥ 2.5 mIU/L (Table 2b,c).

Among all measured linear parameters, blood lymphocyte concentration constituted the only linear variable statistically associated with TSH ≥ 2.5 mIU/L, when evaluated by the univariate regression analysis. Thus, the second step of regression analysis, i.e., the multivariate regression analysis, was not performed, and blood lymphocyte concentration was proven to be the only independent factor associated with TSH ≥ 2.5 mIU/L (Table 3). Similarly, blood lymphocyte concentration constituted the only linear variable statistically associated with TSH ≥ 2.5 mIU/L in the total group of 466 patients when evaluated by the univariate regression analysis (OR = 1.644, *p* < 0.001). However, in the group of 240 patients (with normal thyroid tests but after excluding patients on L-thyroxine), this association of blood lymphocytes and TSH ≥ 2.5 mIU/L lost its statistical significance.

TSH concentration correlated positively with blood lymphocyte concentration (r = 0.129, *p* = 0.031) and with TPOAb concentrations (r = 0.177, *p* = 0.005), and TSH concentration correlated negatively with FT4 concentration (r = −0.157, *p* = 0.009), when the group of patients with normal thyroid tests was considered (n = 280), but not after excluding patients on L-thyroxine treatment.

At the same time, however, no correlations were found between lymphocyte concentration and TPOAb concentration (r = 0.002, *p* = 0.979), between lymphocyte concentration and TgAb concentration (r = −0.022, *p* = 0.740), as well as between lymphocyte concentration and TSHRAb concentration (r = −0.006, *p* = 0.924) in patients with normal thyroid tests (n = 280).

## 4. Discussion

To properly diagnose and treat thyroid dysfunction in women of reproductive age, especially during pregnancy, is of crucial value for public health [16].

The current study was designed on the basis of our previous results to confirm that high-normal TSH is associated with certain unfavorable metabolic changes [14]. Both studies were performed in the same department but they comprised absolutely different groups of patients. The earlier study was a prospective study performed in inpatients hospitalized in 2015, whereas the current study is a retrospective analysis performed in inpatients hospitalized in the period 2016–2017.

In the present study, patients with TSH ≥ 2.5 mIU/L constituted approximately 23–24% of all patients with normal thyroid tests. This percentage is in agreement with what we have observed in our earlier study, i.e., 27.3% [14]. However, it should be remembered that only 5% of absolutely healthy subjects from the general population has TSH in the upper normal range, which obviously results in very low median TSH level. Such a distribution of TSH concentration has been widely accepted in the world and is well confirmed in different studies. For example, in pregnant women, low median TSH has been found in all three trimesters (for example Ekinci et al. [17]).

The above discrepancy concerning the prevalence of high-normal TSH (23–24% vs. 5%) strongly suggests that most of our patients with high-normal TSH are just unhealthy, therefore they require replacement therapy with L-thyroxine. Thus, it seems that there is a huge-scale phenomenon concerning the prevalence of subclinical hypothyroidism in women of childbearing age.

The key finding of our study is the association between high-normal TSH and the abnormal lipid profile in women of childbearing age. As was mentioned in the Introduction, such an association has already been partially documented in the literature. In this context, it is worth mentioning that an abnormal lipid profile during gestation affects unfavorably the course of pregnancy and progeny. For example, high TGs and low HDL cholesterol at late gestation are independent predictors of macrosomia in women without diabetes mellitus [18]. Additionally, it has been documented that higher TSH in normal ranges is strongly associated with a higher BMI in women of childbearing age, thus contributing to obesity [12]. Thus, the current and other observations from the literature related to lipid profiles strongly support the point of view that high-normal TSH in women of reproductive age should be treated as abnormal.

Concerning thyroid antibodies, they occurred more frequently in subjects with TSH ≥ 2.5 mIU/L. Thus, a higher prevalence of thyroid antibodies in patients with high-normal TSH suggests that this group of patients is at very high risk of developing overt hypothyroidism. Again, this observation supports the need for replacement therapy in women with TSH ≥ 2.5 mIU/L. In this context it is worth mentioning that TPOAb positivity has recently been documented to be predictive of a reduced live birth rate in patients with recurrent pregnancy loss; expectedly, L-thyroxine replacement improved live birth rate [19]. TPOAb positivity is also associated with other pregnancy complications, such as preterm birth [3]. In the broader context it is worth adding that thyroid antibodies, i.e., TPOAb and TgAb, were positively associated with the risk of thyroid nodules [20].

A new finding in our study is the association between TSH levels and peripheral blood lymphocyte concentration. Although the absolute number of lymphocytes remained in normal ranges in all subjects, the mean lymphocyte concentration was higher in patients with TSH ≥ 2.5 mIU/L, lymphocyte concentration correlated positively with TSH, and lymphocyte concentration was the only independent factor associated with high-normal TSH. At the same time, lymphocyte concentration was not associated with the level of antibodies. These results suggest that the absolute number of lymphocytes depends directly on thyroid function but it does not depend on autoimmune processes. This positive association between TSH and lymphocyte levels, both remaining in normal ranges, cannot be clearly explained at this moment. However, it can be speculated that this relationship results from an abnormal lipid profile in patients with high-normal TSH. Such a hypothesis can be supported by recently published observations performed concerning patients with type-2 diabetes mellitus in whom proatherogenic parameters of lipid profile correlated positively with the lymphocyte level [21].

It is worth mentioning that different results have been published recently showing an association between high-normal TSH and abnormal values of certain parameters, the significance of which is not clear. For example, it has been observed that serum TSH is positively associated with microalbuminuria in euthyroid diabetic patients and the mechanism of this interesting relationship is currently the subject of speculation [22].

It is well known that reference ranges for thyroid tests depend also on the method used. However, whereas reference intervals for the main thyroid hormone, i.e., thyroxine, are confirmed to be method-related, the reference ranges for TSH do not differ significantly between methods [23]. Taking into account the above cited findings and our results, it is justified to establish a universal cut-off value of 2.5 for the younger population, which can be generally used in different laboratories.

In conclusion, in women of reproductive age with normal thyroid tests, TSH ≥ 2.5 mIU/L is associated with a less favorable lipid profile and with a higher prevalence of thyroid antibodies. These results support our standpoint that high-normal TSH at reproductive ages can be considered abnormal in most patients and, therefore, the replacement therapy with L-thyroxine should be taken into account in such cases, especially with a coexisting abnormal lipid profile or positive thyroid antibodies. Further studies should be performed to find additional benefits from adjusting TSH to the lower end of normal ranges in women of reproductive age. The significance of the positive association between so-called normal TSH levels and normal blood lymphocytes is unknown at this moment and requires further evaluation.

## Figures and Tables

**Table 1:** Mean values (±SEM) of clinical/anthropometric/laboratory parameters in a subgroup with TSH < 2.5 mIU/L and in the subgroup with TSH ≥ 2.5 mIU/L, evaluated in the group of 280 inpatients with normal thyroid tests. Comparison between subgroups was performed by Student’s unpaired *t*-test. Statistical significance was determined at the level of *p* < 0.05. Statistically significant differences are shaded. The level of statistical significance is given in italics. RBC: red blood cells, Hgb: hemoglobin, WBC: white blood cells, FT4: free thyroxine, FT3: free triiodothyronine, TPOAb: thyroid peroxidase antibodies, TgAb: thyroglobulin antibodies, TSHRAb: TSH receptor antibodies, HDLC: HDL cholesterol, LDLC: LDL cholesterol, TGs: triglycerides, CRP: C-reactive protein, ESR: erythrocyte sedimentation rate, IRI: insulin resistance index.

	TSH < 2.5 mIU/L	TSH ≥ 2.5 mIU/L	*p*
Age [years]	29.29 ± 0.60 n = 214	28.18 ± 0.90 n = 66	*0.356*
Body mass [kg]	70.87 ± 1.68 n = 214	73.89 ± 4.03 n = 64	*0.420*
Height [m]	165.56 ± 0.75 n = 214	166.51 ± 0.80 n = 64	*0.527*
BMI [kg/m^2^]	25.66 ± 0.54 n = 155	26.76 ± 1.34 n = 53	*0.365*
RBC [10^12^/L]	4.47 ± 0,.23 n = 213	4.56 ± 0.05 n = 66	*0.093*
Hgb [g/dL]	13.07 ± 0.06 n = 213	13.29 ± 0.12 n = 66	*0.113*
WBC [10^9^/L]	7.07 ± 0.13 n = 212	7.16 ± 0.25 n = 66	*0.745*
Neutrophils [10^9^/L]	3.80 ± 0.12 n = 212	3.65 ± 0.19 n = 66	*0.541*
Lymphocytes [10^9^/L]	2.49 ± 0.047 n = 212	2.720 ± 0.095 n = 66	*0.023*
Platelets [10^9^/L]	255.15 ± 3.89 n = 200	260 ± 7.01 n = 65	*0.464*
FT4 [ng/dL]	1.22 ± 0.01 n = 214	1.20 ± 0.02 n = 66	*0.327*
FT3 [pg/mL]	3.09 ± 0.02 n = 199	3.10 ± 0.04 n = 61	*0.981*
TPOAb [IU/mL]	29.51 ± 5.60 n = 186	67.49 ± 14.77 n = 62	*0.004*
TgAb [IU/mL]	48.27 ± 12.62 n = 180	86.88 ± 23.57 n = 60	*0.135*
TSHRAb [IU/L]	0.25 ± 0.02 n = 176	0.36 ± 0.05 n = 56	*0.054*
Cholesterol [mg/dL]	170.02 ± 2.18 n = 200	168.94 ± 3.66 n = 65	*0.803*
HDLC [mg/dL]	54.99 ± 1.02 n = 200	54.07 ± 1.95 n = 65	*0.667*
LDLC [mg/dL]	93.15 ± 1.97 n = 200	92.81 ± 3.78 n = 65	*0.934*
HDLC/Cholesterol	0.33 ± 0.006 n = 200	0.33 ± 0.01 n = 64	*0.963*
TGs [mg/dL]	96.31 ± 4.11 n = 199	105.40 ± 8.66 n = 65	*0.298*
Glucose [mg/dL]	83.66 ± 0.70 n = 198	83.20 ± 1.50 n = 60	*0.762*
CRP [mg/dL]	0.35 ± 0.08 n = 76	0.26 ± 0.14 n = 16	*0.647*
ESR	11.13 ± 1.49 n = 53	9.84 ± 2.07 n = 19	*0.644*
IRI	1.11 ± 0.03 n = 111	1.07 ± 0.05 n = 39	*0.503*
Vit D [ng/mL]	20.50 ± 0.62 n = 170	21.89 ± 1.29 n = 53	*0.293*
Iron [µg/dL]	83.0 ± 6.35 n = 27	90.50 ± 10.29 n = 10	*0.542*

**Table:** (**a**)

	TSH < 2.5 mIU/L, n = 301	TSH ≥ 2.5 mIU/L n = 111	*p*
TPOAb ≥34 IU/mL	n = 42, 14%	n = 41, 37%	*0.001*
TgAb ≥115 IU/mL	n = 45, 15%	n = 31, 28%	*0.003*
TSHRAb ≥1.75 IU/L	n = 15, 5%	n = 2, 2%	*0.178*

**Table:** (**b**)

	TSH < 2.5 mIU/L n = 186	TSH ≥ 2.5 mIU/L n = 62	*p*
TPOAb ≥34 IU/mL	n = 17, 9%	n = 15, 27%	*0.001*
TgAb ≥115 IU/mL	n = 18, 10%	n = 9, 16%	*0.201*
TSHRAb ≥1.75 IU/L	n = 1, 1%	n = 0, 0%	*-*

**Table:** (**c**)

	TSH < 2.5 mIU/L n = 134	TSH ≥ 2.5 mIU/L n = 45	*p*
TPOAb ≥34 IU/mL	n = 9, 7%	n = 9, 20%	*0.013*
TgAb ≥115 IU/mL	n = 12, 9%	n = 5, 11%	*0.692*
TSHRAb ≥1.75 IU/L	n = 1, 1%	n = 0, 0%	*-*

**Table 3:** Univariate logistic regression analysis of the univariate increased TSH (for TSH ≥ 2.5 mIU/L) determinants (variables), performed in women of childbearing age with normal thyroid tests (n = 280). OR, odds ratio; CI, confidence interval; Statistical significance was determined at the level of *p* < 0.05. Statistically significant differences are shaded. The level of statistical significance is given in italics.

Variable	Univariate Regression		
	OR	95% CI	*p*; n
Age [years]	0.985	0.95–1.02	*p = 0.355*; n = 280
Body mass [kg]	1.005	0.99–1.01	*p = 0.420*; n = 199
Height [m]	1.014	0.97–1.06	*p = 0.526*; n = 198
BMI [kg/m^2^]	1.018	0.97–1.05	*p = 0.364*; n = 208
RBC [10^12^/L]	1.921	0.89–4.13	*p = 0.093*; n = 279
Hgb [g/dL]	1.265	0.94–1.69	*p = 0.114*; n = 279
WBC [10^9^/L]	1.023	0.89–1.17	*p = 0.744*; n = 278
Neutrophils [10^9^/L]	0.948	0.80–1.12	*p = 0.540*; n = 278
Lymphocytes [10^9^/L]	1.551	1.05–2.27	*p = 0.024*; n = 278
Platelets [10^9^/L]	-	-	-
FT4 [ng/dL]	0.414	0.07–2.43	*p = 0.327*; n = 280
FT3 [pg/mL]	1.009	0.42–2.39	*p = 0.982*; n = 260
TPOAb [IU/mL]	-	-	-
TgAb [IU/mL]	-	-	-
TSHRAb [IU/L]	1.012	0.98–1.04	*p = 0.383*; n = 234
Cholesterol [mg/dL]	0.998	0.99–1.01	*p = 0.802*; n = 265
HDLC [mg/dL]	0.995	0.97–1.01	*p = 0.665*; n = 265
LDLC [mg/dL]	0.999	0.99–1.01	*p = 0.933*; n = 265
HDLC/Cholesterol	1.072	0.05–21.63	*p = 0.963*; n = 264
TGs [mg/dL]	-	-	-
Glucose [mg/dL]	0.995	0.97–1.02	*p = 0.761*; n = 258
CRP [mg/dL]	0.807	0.32–2.03	*p = 0.644*; n = 92
ESR	0.986	0.93–1.04	*p = 0.640*; n = 72
IRI	0.696	0,24–1.05	*p = 0.500*; n = 150
Vit D [ng/mL]	1.019	0.98–1.05	*p = 0.292*; n = 223
Iron [µg/dL]	1,007	0.98–1.03	*p = 0.531*; n = 37

## Abbreviations

CRP—C-reactive protein

MBL—Mannan Binding Lectin

TSH—thyroid-stimulating hormone

FT4—free thyroxine

FT3—free triiodothyronine

IRI—insulin resistance index

TPOAb—thyroid peroxidase antibodies

TgAb—thyroglobulin antibodies

TSHRAb—thyrotropin receptor antibodies

ELISA—enzyme-linked immunosorbent assay

HDL—high-density lipoprotein

LDL—low-density lipoprotein

TG—triglycerides

LPO—lipid peroxidation
